# The interactome of tau phosphorylated at T217 in Alzheimer’s disease human brain tissue

**DOI:** 10.1007/s00401-025-02881-8

**Published:** 2025-05-03

**Authors:** Tomas Kavanagh, Manon Thierry, Kaleah Balcomb, Jackeline Ponce, Evgeny Kanshin, Alexander Tapia-Sealey, Glenda Halliday, Beatrix Ueberheide, Thomas Wisniewski, Eleanor Drummond

**Affiliations:** 1 Neuroscience, School of Medical Sciences, Faculty of Medicine and Health, University of Sydney, Camperdown, NSW 2050 Australia; 2 Brain and Mind Centre, University of Sydney, Camperdown, NSW 2050 Australia; 3 Center for Cognitive Neurology, New York University Grossman School of Medicine, New York, NY 10016 USA; 4 Department of Neurology, New York University Grossman School of Medicine, New York, NY 10016 USA; 5 Proteomics Laboratory, Division of Advanced Research Technologies and Department of Biochemistry and Molecular Pharmacology, New York University Grossman School of Medicine, New York, NY 10016 USA; 6 Department of Biochemistry and Molecular Pharmacology, New York University Grossman School of Medicine, New York, NY 10016 USA; 7 Department of Pathology, New York University Grossman School of Medicine, New York, NY 10016 USA; 8 Department of Psychiatry, New York University Grossman School of Medicine, New York, NY 10016 USA

**Keywords:** Alzheimer’s disease, Tau, T217, ApoE, Interactome, CTLH E3 ubiquitin ligase, Proteomics, p62

## Abstract

Hyperphosphorylated tau (pTau) in Alzheimer’s disease (AD) brain tissue is a complex mix of multiple tau species that are variably phosphorylated. The emerging studies suggest that phosphorylation of specific residues may alter the role of tau. The role of specific pTau species can be explored through protein interactome studies. The aim of this study was to analyse the interactome of tau phosphorylated at T217 (pT217), which biomarker studies suggest is one of the earliest accumulating tau species in AD. pT217 interactors were identified in fresh-frozen human brain tissue from 10 cases of advanced AD using affinity purification-mass spectrometry. The cases included a balanced cohort of *APOE* ε3/ε3 and ε4/ε4 genotypes (*n* = 5 each) to explore how apolipoprotein E altered phosphorylated tau interactions. The results were compared to our previous interactome dataset that profiled the interactors of PHF1-enriched tau to determine if individual pTau species have different interactomes. 23 proteins were identified as *bona fide* pT217 interactors, including known pTau interactor SQSTM1. pT217 enriched tau was phosphorylated at fewer residues compared to PHF1-enriched tau, suggesting an earlier stage of pathology development. Notable pT217 interactors included five subunits of the CTLH E3 ubiquitin ligase (WDR26, ARMC8, GID8, RANBP9, MAEA), which has not previously been linked to AD. In *APOE* ε3/ε3 cases pT217 significantly interacted with 46 proteins compared to 28 in *APOE* ε4/ε4 cases, but these proteins were significantly overlapped. CTLH E3 ubiquitin ligase subunits significantly interacted with phosphorylated tau in both *APOE* genotypes. pT217 interactions with SQSTM1, WDR26 and RANBP9 were validated using co-immunoprecipitation and immunofluorescent microscopy of post-mortem human brain tissue, which showed colocalisation of both protein interactors with tau pathology. Our results report the interactome of pT217 in human Alzheimer’s disease brain tissue for the first time and highlight the CTLH E3 ubiquitin ligase complex as a significant novel interactor of pT217 tau.

**Supplementary Information:**

The online version contains supplementary material available at 10.1007/s00401-025-02881-8.

## Introduction

Hyperphosphorylation and accumulation of the protein tau is a hallmark of Alzheimer’s disease (AD) [20, 50]. Phosphorylation appears to occur in a stepwise fashion and contributes to the dysfunction and aggregation of tau in disease [1, 2, 32, 40, 60, 64]. The phosphorylation of specific residues of tau can both act as biomarkers for AD and track progression of the disease [9, 35, 46, 53]. Fluid biomarker studies suggest that tau phosphorylated at threonine 217 (pT217) is one of the earliest accumulating pathological tau species in AD [8, 10, 24, 36, 38, 53] and can accurately predict amyloid pathology before the development of cognitive symptoms. pT217 increases with AD progression and discriminates AD from other tauopathies [8, 35, 42, 46]. High pT217 concentrations in cerebrospinal fluid (CSF) is able to predict the likelihood of conversion of individuals from normal cognition to mild cognitive impairment and from mild cognitive impairment to AD [46]. Whilst pT217 is dysregulated early in disease and is an excellent disease biomarker, many questions remain about the pathological role of pT217 tau in the brain in AD.

The study of protein interactions with tau have revealed important insights into disease processes [11, 17, 21, 22, 25, 27, 31, 48, 52, 54, 57–59, 63]. Some of these insights include the significant interaction between RNA binding proteins and tau that may amplify the aggregation and toxicity of pathological tau [25, 57] and that interactions between tau and components of ubiquitin-dependent proteolysis pathways are important for tau degradation and preventing tau aggregation [7, 44, 62]. Most studies of tau interactions in the human brain to date have focussed on interactions of all tau species pooled together [6, 22, 37] or heavily phosphorylated tau species that peak later in disease (such as PHF1 which detects tau phosphorylated at S396/S404) [17, 52]. To date, no studies have examined the interactome of earlier accumulating tau species in AD such as pT217. Here we perform affinity purification mass spectrometry (AP-MS) on post-mortem AD brain tissue with antibodies targeting the pT217 epitope to determine if these interactions vary from more ‘mature’ phosphorylated tau species and can highlight potential early interactions related to AD pathogenesis. These studies were performed in the same cohort of human brain samples segregated by *APOE* genotype used in our previous study assessing the PHF1-immunoreactive phosphorylated tau interactome [52], thus permitting the direct comparison of the interactome of two phosphorylated tau (pTau) species and the examination of the effect of *APOE* genotype on the pT217 interactome.

## Materials and methods

### Ethics statement

All procedures were performed under protocols approved by Institutional Review Boards at New York University Alzheimer Disease Research Centre, NY (USA) and Sydney University Human Ethics Committee (Australia). In all cases, written informed consent for research was obtained from the patient or legal guardian and the material used had appropriate ethical approval for use in this project. All patients’ data and samples were coded and handled according to NIH guidelines to protect patients’ identities. The validation experiments were performed on tissue acquired from the Sydney Brain Bank. Post-mortem brain tissue use was approved by the Sydney Institutional Ethics Committee.

### Patient tissue selection

A total of 20 cases of sporadic AD were included in this study alongside seven cognitively normal controls, two corticobasal degeneration cases, 1 Pick’s disease case, and 1 progressive supranuclear palsy case. The cases were selected from donated brain tissue collected at the NYU ADRC and Sydney Brain Bank, based on their ABC score (A3, B3, C2-3), severity of tau pathology in the frontal cortex, and *APOE* genotype. The *APOE* ε3/ε3 and *APOE* ε4/ε4 cases used for AP-MS experiments were matched to the best of our ability in terms of age, sex and co-morbidities, as shown in Supplementary Table 1. Our inclusion criteria involved the absence of any additional primary tauopathy and of any major co-pathology. Individual case information is detailed in Supplementary Table 1. The genotypes were provided by NYU Alzheimer Disease Research Centre as described previously [52].

### Tissue homogenisation

Tissue homogenisation was performed using our previously published methods [17]. Briefly, 250 mg of grey matter was dissected from frozen tissue blocks of each sample. The tissue was pulverised on dry ice with a hammer. Pulverised tissue was homogenised in low salt homogenisation buffer [50 mM HEPES pH 7.0, 250 mM Sucrose, 1 mM EDTA, protease inhibitor cocktail (cOmplete mini tablets, EDTA-free Millipore Sigma) and phosphatase inhibitor cocktail (PhosSTOP EASYpack, Roche)] with a dounce homogeniser. Protein concentration was determined using a Micro BCA assay kit (Thermo Micro BCA Assay Cat. No.: 23235). Homogenised samples were flash frozen in a dry ice:ethanol slurry and stored at − 80 °C.

### Western blotting

Western blot was used to profile cases for phosphorylated tau epitopes and confirm that co-immunoprecipitation successfully enriched tau. For total homogenate western blots, 15 μg of total protein was mixed with Bolt LDS sample buffer, 10 mM DTT and boiled for 5 min. For validation IPs, the total output was boiled in 1X LDS sample loading buffer and 10 mM DTT for 5 min. For AP-MS experiments 10 per cent of the output elution was mixed with Bolt LDS sample buffer and 10 mM DTT and boiled for 5 min. The proteins were then run on 4–12% NuPage Bis–Tris 26-well mini and midi gels (Invitrogen). The proteins were then transferred to 0.2 µM nitrocellulose then blocked with 5% skim milk or 5% BSA in TBS-T. Blots were probed overnight at 4 °C with PHF1 (pS393/S404; provided by Professor Peter Davies, 1:500), pT217 tau (Thermo, #44-744, 1:1000), pT231 tau (Thermo, AT180 #MN-1040, 1:1000), pTau S199/202 (Thermo, #44-768G, 1:500), total tau (Abcam, rabbit ab76128, mouse ab80579, 1:1000 or Thermo, MA5-12808 1:1000), p62/SQSTM1 (Abcam, ab56416 1:000), RANBP9 (Abcam, ab52605, 1:1000) or WDR26 (Abcam, ab85962, 1:500). The specificity of the tau and WDR26 antibodies has been confirmed in previous studies [18, 41]. The blots were then incubated for 2 h at room temperature with secondary antibody (anti-rabbit or anti-mouse HRP conjugated antibody, 1:3000). The western blots were then developed with ECL western blotting substrate (Pierce #32106, Merck #WBULS0500) imaged on a BioRad ChemiDoc imaging system or Thermo CL1500 gel imaging system.

### Co-immunoprecipitation

Co-immunoprecipitation (co-IP) of tau phosphorylated epitopes was performed on all cases with tau pT217 (Thermo, #44-744) and a complimentary rabbit (Thermo, #02-6102) control IgG as previously described [17]. We also attempted to use the same approach to study the pT231 interactome using a pT231 antibody (AT180, Thermo, #MN1040) and a mouse IgG control antibody (Biolegend #400102), however, pT231 immunoprecipitation did not enrich tau sufficiently to analyse the interactome at our desired stringency. We have included these results in supplementary data, in case they are of interest to the field. Each IP used a total of 1.5 mg of total protein from homogenised tissue and 10 µg of each antibody. Brain homogenates were incubated overnight with antibody with end-over-end rotation. Protein G DynaBeads (7.5 mg/sample) were washed three times prior to sample addition in Citrate Phosphate wash buffer (pH 5.0, 25 mM citric acid, 65 mM Dibasic sodium phosphate, 0.01% Tween-20). The samples were then incubated with protein G DynaBeads overnight at 4 °C with end-over-end rotation. Bound beads were recovered with a magnet and washed three times with Citrate Phosphate buffer (pH 5.0, 25 mM citric acid, 65 mM dibasic sodium phosphate). 10% of IP product was eluted from beads in denaturing conditions (Bolt LDS sample buffer, 75 °C, shaking for 15 min) and 5% used for WB validation. The remaining antigen:antibody bound beads were stored in PBS at 4 °C until use for AP-MS. For validation Co-IPs, 300 µg total protein from brain homogenates was combined with 2 µg antibody (tau pT217 Thermo #44-744, total Tau Abcam ab76128, total Tau Thermo #MA5-12808, mouse IgG isotype control Biolegend #400102 or rabbit IgG isotype control Thermo #02-6102) in a total volume of 350 µL. The samples were processed as described above but the total output was eluted with denaturing conditions and used for western blotting to confirm selected interactions.

### Sample preparation for MS analysis: on-bead digestion

Proteins enriched via co-IP were reduced with DTT (20 mM) at 57 °C for 1 h and subsequently alkylated with iodoacetamide (45 mM) at room temperature in the dark for 45 min. Antigen:antibody complexes were then digested on the beads with sequencing grade modified trypsin (Promega) overnight at room temperature with shaking. The digested samples were the acidified with 10% TFA (final concentration 0.5%) and loaded onto equilibrated Pierce C18 Spin columns. The bound samples were then washed with 0.1% trifluoroacetic acid followed by 0.5% acetic acid. The samples were eluted with 80% acetonitrile in 0.5% acetic acid. Organic solvents were removed in a SpeedVac concentrator and reconstituted in 0.5% acetic acid.

### LC MS/MS

An aliquot of each peptide mixture was analysed for each sample. Liquid chromatography (LC) separation was performed online with mass spectrometry (MS) using the autosampler EASY-nLC 1000 (Thermo Scientific). Peptides were gradient eluted from the column directly to the Orbitrap Eclipse with a 1 h gradient (Thermo Scientific, Solvent A 2% acetonitrile in 0.5% acetic acid, Solvent B 80% acetonitrile in 0.5% acetic acid).

Full MS spectra were acquired with a high resolution of 120,000, an AGC target of 4e5, a maximum ion time of 50 ms and a scan range of 400–1500 *m*/*z*. All MS/MS spectra were collected using the following instrument parameters: resolution of 30,000, AGC target of 2e5, maximum ion time of 200 ms, one microscan, 2 *m*/*z* isolation window and NCE of 27.

### Immunohistochemistry and imaging of formalin fixed paraffin embedded human brain tissue

Formalin-fixed paraffin-embedded tissue sections underwent fluorescent immunohistochemistry using the method described in [16]. Briefly, the sections were deparaffinized and rehydrated through a series of xylene and ethanol washes. Antigen retrieval was achieved by boiling in citrate buffer for 21 min (10 mM sodium citrate, 0.05% Tween-20, pH 6). Sections were blocked in 10% normal goat serum for 1 h and incubated with anti-SQSTM1 (Abcam, ab56416 1:100), or anti-WDR26 (Abcam, ab85962, 1:100), combined with either pT217 tau (Thermo, #44-744, 1:500), pT231 tau (Thermo, AT180 #MN-1040, 1:500) and anti-pTau (ThermoFisher, AT8, MN1020, 1:500) in 4% normal goat serum overnight at 4 °C. AlexaFluor488-, AlexaFluor647-conjugated secondary antibodies (Jackson ImmunoResearch Laboratories, 1:500) and Hoechst 33342 (Sigma, B2261, 1:500) were applied for 2 h at room temperature prior to coverslipping with Antifade ProLong Glass (Invitrogen, P36984). For each batch of staining a single secondary only slide was used as a negative control. Whole slide images were acquired using an Olympus VS200 Slide Scanner at 20× magnification. Representative 60× images (NA 1.4) were captured on a Nikon C2 Confocal microscope. Empty channel *λ*568 was captured at equal settings to channel *λ*488 to allow for autofluorescence subtraction in ImageJ. Images are evenly adjusted per channel in Photoshop for presentation purposes.

### Proximity ligation assays (PLA)

FFPE tissue sections were prepared for PLA as detailed above. Briefly, the sections were deparaffinized and rehydrated through a series of xylene and ethanol washes. Antigen retrieval was achieved by boiling in citrate buffer for 21 min (0.05 mM sodium citrate, 0.05% Tween-20, pH 6). Sections were blocked in 10% normal horse serum and incubated with anti-WDR26 (Abcam, ab85962, 1:100), anti-AT8 (Thermo, MN1020, 1:2000) and anti-tau (R&D Systems, AF3494, 1:1000) in 4% normal horse serum overnight at 4 °C. Proximity ligation was performed by incubating slides with Duolink anti-mouse PLUS and anti-rabbit MINUS probes (Sigma-Aldrich, DUO92001, DUO92005) followed by detection reagents (Sigma-Aldrich, DUO92013) according to the manufacturer’s protocol. AlexaFluor488-conjugated donkey anti-goat secondary (Jackson, 705-545-147, 1:1000) and Hoechst 33342 (Sigma, B2261, 1:500) were then applied for 1.5 h at room temperature prior to coverslipping with Antifade ProLong Glass (Invitrogen, P36984). For a negative control, one section was treated with secondary antibodies only. The whole slide images were acquired using an Olympus VS200 Slide Scanner at 20× (NA 0.8) magnification. Representative 60× (NA 1.4) images were captured on a Nikon C2 Confocal microscope. Empty channel *λ*568 was captured at equal settings to channel *λ*647 to allow for autofluorescence subtraction in ImageJ. Images are evenly adjusted per channel in Photoshop for presentation purposes.

### Data analysis

The MS/MS spectra were searched against a Uniprot human database using Sequest within Proteome Discoverer 1.4. Variable modification of oxidation on methionine, deamidation on glutamine and asparagine, phosphorylation on serine, threonine and tyrosine, and a fixed modification of carbamidomethylation on cysteine was enabled. The data were filtered to better than 1% peptide and protein FDR searched against a decoy database. Only proteins with at least two different peptides were considered for downstream analysis.

To determine the most likely tau pT217 and pT231 interactors (compared to rabbit IgG or mouse IgG control respectively), we performed a SAINT Express analysis on this data set as previously detailed for both whole of epitope and *APOE* genotype interactors (Supplementary Tables 2–8) [12, 17, 51]. We considered a protein a bona fide interactor if it had a SAINT score ≥ 0.65 [17]. The common contaminants were removed from the analysis. For the phospho epitope analysis we searched the data using Andromeda within the Maxquant platform [13, 14] including the PHF1 IP data from [52] to be able to compare and contrast the identified phosphosites. The contaminant database was concatenated with Tau isoform F and the data filtered to better than 1% FDR using a decoy database.

**Fig. 1:**
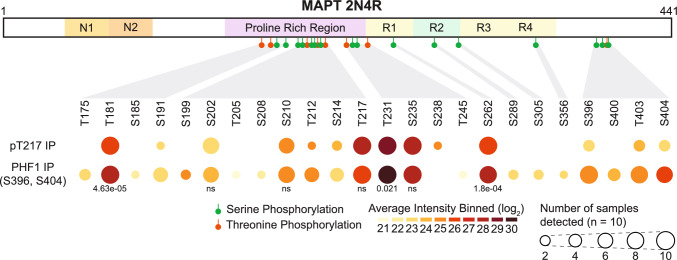
Phosphorylation profiles of tau enriched by pT217 and PHF1 co-IP. Diagram mapping location and intensity of phosphorylated residues detected in co-IPs of pT217 and PHF1 tau (S396, S404) mapped against full length 2N4R tau. Green flags represent serine phosphorylation and orange represent threonine phosphorylation. Colour shade of nodes represents the binned average intensity (i.e. 21 = 21–21.99, 30 = 29–30) for each residue. Node size correlated to the number of samples positive for phosphorylation. Statistics shown are group-wide results. *ns* not significant

To normalise intensities for the detected tau phosphosites across different IPs, we summed the intensity for five different non-phosphorylated Tau peptides that are quantified across all the experiments as a proxy for the total Tau protein quantity. The MS intensities for the phosphosites are then normalised to the total Tau quantity. This allows us to characterise the different phosphorylation patterns characteristic for each enriched epitope (as shown on Fig. 1, Supplementary Table 9).

### Bioinformatics and statistics

General data analysis and plotting was performed in R Studio (R v4.3.2) with the following packages: tidyverse 2.0.0, ggpubr 0.6.0. Gene ontology over-representation analysis was performed in R with the clusterProfiler package (v4.8.3) with the human database Org.Hs.eg.db v3.17.0 and enrichment plots made with enrichplot v1.20.3. Large gene ontology over representation lists were shortened for enrichment maps using the ‘simplify’ function in enrichplot to remove term descriptions with more than 70% similarity. The intersection statistics were performed with the geneOverlap package v1.36.0 which uses Fisher’s exact test to find the significance, odds ratio and Jaccard index for overlapping lists. Intersection stats were generated for comparisons between pT217 interactors in *APOE* genotypes, total pT217 interactors and PHF1 interactors published previously [52]. The venerable package v3.1.0.9000 was used to compute Venn diagrams. Spearman’s rank correlation was used to correlate the lists of tau interactors for each co-IP and *APOE* genotypes, using Saint Score to order the gene lists. STRING 11.5 and Cytoscape 3.9.1 was used to construct networks of tau interactors with a high confidence cut-off of 0.7 for known interactions. Biological groups were manually assigned based on strings annotations. Figures were created in Adobe Photoshop 26.0 and Illustrator 28.1.

## Results

### pT217 co-immunoprecipitation enriches for tau species with fewer phosphorylated residues

To characterise the phosphorylation profiles of the tau species enriched by co-IP using different pTau-targeting antibodies, we compared the abundance of phosphorylated tau residues identified after co-IP of pT217 and pT231 and pS396/S404 (PHF1) in the same 10 cases of advanced AD (Supplementary Table 9). PHF1 co-IP detected the most phosphorylated residues (23 sites in at least one case), while pT217 co-IP detected 14 sites (Fig. 1). The phosphorylation of serine residues 202, 210, 235 and 262 as well as threonine 181, 217, and 231 were common across all co-IPs. All phosphorylated residues were most intense in PHF1 enriched tau except for pT217. Phosphorylation of T217 residues was detected across all samples but had the highest average intensity in T217 co-IP, although there was no significant difference between groups (LFQs; PHF1 co-IP = 26.1, pT217 co-IP = 27.0, ANOVA *F* value = 0.83, *p*-value = 0.447). Similarly, phosphorylation of T231 residues was detected across all samples but had the highest average intensity in PHF1 co-IP (LFQs; PHF1 co-IP = 29.2, pT217 co-IP = 28.3, Kruskal–Wallis test *χ*^2^ = 7.714, *p* = 0.02). Phosphorylation of serine 396 and 404, the PHF1 epitope, was strongly detected in PHF1 co-IPs but only weakly detected in pT217 co-IPs (LFQs = 22.8 and 23.0 respectively). pT181 and pS262 were also detected in all samples with the greatest intensities in PHF1 enriched tau (pT181; ANOVA *F* = 14.77, *p*-value = 4.63 × 10^–5^, pS262 Kruskal–Wallis test *χ*^2^ = 17.299, *p* = 1.8 × 10^–4^). Overall, pT217 enriched tau had less phosphorylated residues and a lower average intensity of phosphorylation of these residues than PHF1 enriched tau, with the notable exception of T217 (Fig. 1, Supplementary Table 9). These results clearly demonstrated that different pTau antibodies enriched pools of tau with differing phosphorylation profiles and validated the ability of pT217 and PHF1 antibodies to enrich tau phosphorylated at residues T217 and S396/S404 respectively in comparison to other antibodies.

### Epitope specific IP highlights multiple tau protein degradation pathways

**Fig. 2:**
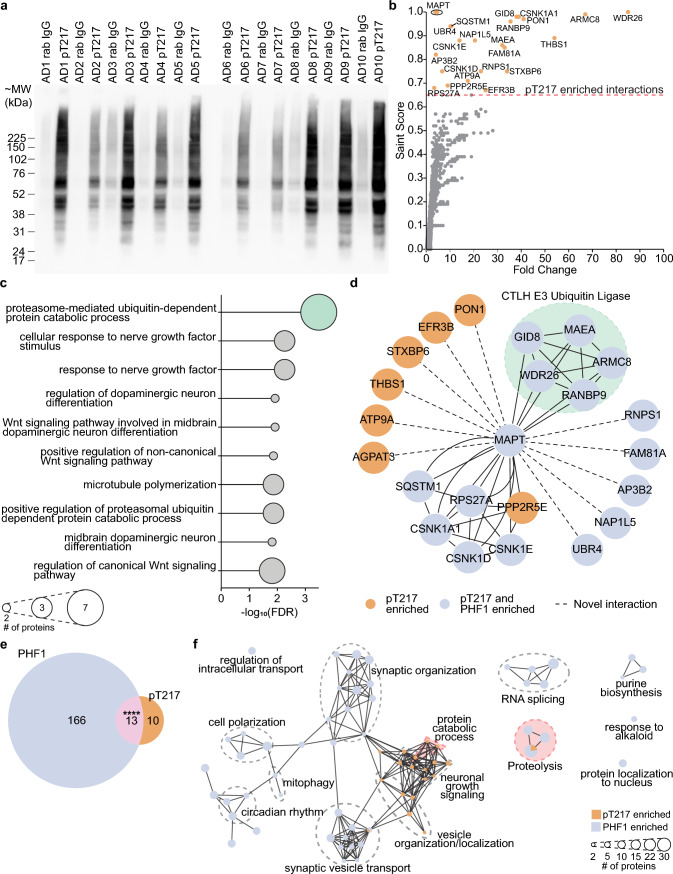
pT217 co-immunoprecipitation highlights the CTLH E3 ubiquitin ligase as a key interactor. **a** Western blot of pT217 co-IP demonstrated strong enrichment for phosphorylated tau at T217 in comparison to control IgG co-IP (*n* = 10 AD cases: *n* = 5 *APOE* ε3/ε3 and *n* = 5 *APOE* ε4/ε4). **b** pT217 tau interactors identified by mass-spectrometry (SAINT score ≥ 0.65). **c** Top 10 over-represented gene ontology biological process terms for the 23 pT217 interacting proteins, sorted by FDR. **d** Protein–protein interaction network highlighted two strongly interacting clusters of proteins. Dotted lines represent novel interactions newly identified in this study not annotated in string.db (https://string-db.org/). Blue nodes highlight proteins that interact with both pT217 and PHF1-immunoreactive pTau, whilst orange nodes highlight proteins that interact with pT217 tau and not PHF1-immunoreactive pTau. **e** Venn diagram depicting the overlap between PHF1-immunoreactive pTau and pT217 tau interactors. Fisher’s exact test, *p* value = 9 × 10^–23^. **f** Enrichment map of common and unique PHF1 [52] and pT217 over-represented gene ontology biological processes. Raw IP and input total homogenate WB images are in Supplementary Fig. 3

To determine if the phosphorylation of tau at residues T217 and T231 altered the interactome of tau, we performed epitope specific co-immunoprecipitation of AD frontal cortex (*n* = 10). AP-MS of pT217 detected a total of 3,325 proteins with 139 enriched compared to control IgG co-IPs (FC > 1.5, *p*-value < 0.05). Tau was the most significantly enriched protein after pT217 tau co-IP (SAINT score = 1.0, FC = 3.83–6.86, Supplementary Table 3, Fig. 2a). pT231 co-IP detected 3,342 proteins, with 516 proteins enriched compared to control IgG co-IPs (FC > 1.5, *p*-value < 0.05). However, while high levels of tau were detected in pT231 co-IP, it was not significantly enriched in comparison to control IgG co-IPs (SAINT score = 0.2–0.22, FC = 2.94–3.09, Supplementary Fig. 2), suggesting that this antibody was less effective at enriching pTau via co-IP in comparison to pT217 or PHF1. The poorer performance of pT231 for co-IP was also apparent on western blot (Supplementary Fig. 2a). Given this, pT231 co-IP results were not included in the interactome analysis below but results can be viewed in Supplementary Fig. 1, 2, 3 and 4 and Supplementary Tables 4, 7, 8, 10, 11.

pT217 co-IP detected 23 *bona fide* pT217 protein interactors based on a SAINT score ≥ 0.65 (Fig. 2b, Supplementary Table 3). The top hits included WDR26, ARMC8, CSNK1A1, GID8 and MAPT (SAINT score = 1, Fig. 2b). The pT217 interacting proteins enriched for gene ontology processes involved in protein catabolism (ubiquitin-dependent) and neuronal growth factor response elements (Fig. 2c). Protein–protein interaction networks of these proteins revealed two separate hubs of proteins involved in protein catabolism. This included a p62/SQSTM1 hub and a CTLH E3 ubiquitin ligase hub (Fig. 2d, Supplementary Table 3). There was significant overlap with our previously published PHF1-immunoreactive pTau interactomes, with 13 of these proteins also interacting with PHF1-immunoreactive pTau reported in our previous study (Fig. 2d, e, Fisher’s exact test *p*-value = 9 × 10^–23^) [52]. These overlapping proteins shared functions in proteolysis, but interactors of pT217 tau were fewer and enriched more strongly for processes involved in protein catabolism, neuronal outgrowth, and vesicle organisation (Fig. 2f, Supplementary Table 12). Despite significant overlap, the interactomes of pT217 and PHF1 tau were only weakly correlated (Spearman’s rho = 0.303, *p*-value 2.00 × 10^–46^) suggesting the enrichments for most interactors varied between co-IP experiments. Interestingly, pT217 co-IP did not enrich several pathways that were strongly enriched in the PHF1-immunoreactive pTau interactome such as RNA splicing, synaptic organisation or vesicle transport (Fig. 1f, Supplementary Table 13).

### APOE genotype effect on pT217 tau interactions

**Fig. 3:**
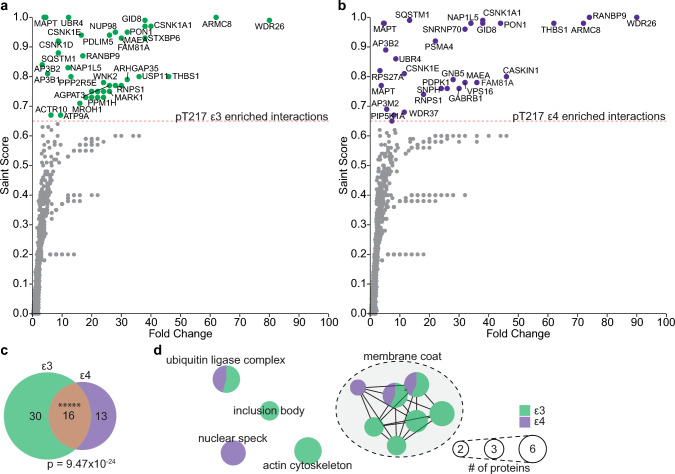
pT217 interactors enriched in APOE ε3/ε3 or APOE ε4/ε4 patients. **a** pT217 tau interactors identified in ε3/ε3 patients by mass-spectrometry (SAINT score ≥ 0.65) of pT217 enriched fractions. **b** pT217 tau interactors identified in ε4/ε4 patients by mass-spectrometry (SAINT score ≥ 0.65) of pT217 enriched fractions. **c** Overlap of pT217 interactors enriched in *APOE* ε3/ε3 and ε4/ε4 patients, Jaccard index = 0.28, Fisher’s exact test, *p* = 4.28 × 10^–24^. **d** Enrichment map of gene ontology cellular compartments over-represented from pT217 interactors in *APOE* ε3/ε3 or ε4/ε4 sub-groups (Supplementary Table 14)

To determine if *APOE* genotype alters the tau pT217 interactions, we performed a sub-analysis of interacting proteins in the *n* = 5 *APOE* ε3/ε3 and *n* = 5 *APOE* ε4/ε4. 46 and 29 proteins significantly interacted with tau phosphorylated at T217 in *APOE* ε3/ε3 cases and *APOE* ε4/ε4 cases respectively (SAINT score ≥ 0.65, Fig. 3a, b). There was significant intersection between *APOE* genotype-specific tau interactors with 16 proteins in common (Fig. 3c, Jaccard Index = 0.28, Fisher’s exact test *p*-value = 9.47 × 10^–24^). Notably, the proteins in common between both genotypes included the five components of the CTLH E3 ubiquitin ligase and related proteins (UBR4, SQSTM1) identified in our combined analysis. Ranked correlation between all identified proteins in *APOE* ε3/ε3 and *APOE* ε4/ε4 subsets of pT217 tau co-IPs was high (Spearman’s rho = 0.77, *p*-value ~ 0) suggesting that interaction with pT217 tau were predominantly the same between genotypes. Most instances of extreme differences in SAINT score were in proteins detected at low abundance.

### Validation of SQSTM1, WDR26 and RANBP9 interactions with tau

We validated the interaction between pT217 tau, and three key proteins linked to ubiquitin-mediated protein degradation and autophagy: SQSTM1 (also referred to as p62), WDR26 and RANBP9. All proteins were highly significant and consistent pT217 tau interactors. First, we performed co-IPs in a second independent cohort consisting of three AD cases, three control cases and one case each of three primary tauopathies (corticobasal degeneration [CBD], Pick’s disease [PiD] and progressive supranuclear palsy [PSP]).The primary tauopathies were included to determine if these protein–protein interactions were AD-specific. Second, immunofluorescent staining of human brain sections was performed in a third independent cohort consisting of three AD cases and three control cases for SQSTM1 and WDR26. Third, we performed a proximity ligation assay (PLA) to more specifically demonstrate the proximity of WDR26 and phosphorylated tau.

**Fig. 4:**
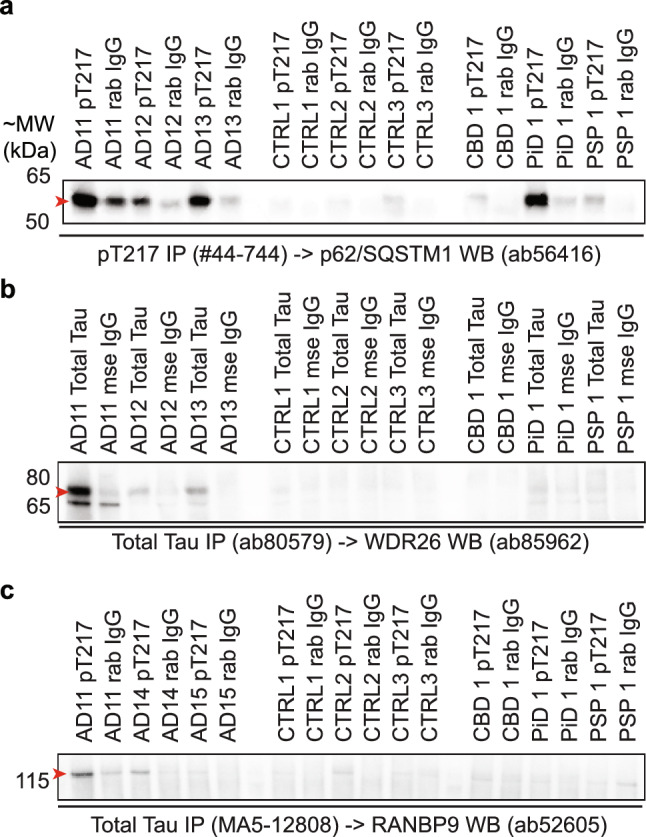
p62/SQSTM1, WDR26 and RANBP9 interact with tau in independent validation cohorts. **a** Western blot for p62/SQSTM1 following pT217 IP in a second cohort of AD patients, controls and tauopathy patients. **b** Western blot for WDR26 following total tau IP in a second cohort of AD patients, controls and tauopathy patients. **c** Western blot for RANBP9 following total tau IP. Samples include post-mortem brain tissue from *n* = 3 AD, *n* = 3 no pathology controls, *n* = 1 Corticobasal degeneration, *n* = 1 Pick’s disease and *n* = 1 progressive supranuclear palsy patients. Arrows indicate the specific band for p62/SQSTM1 at ~ 55 kDa, WDR26 at ~ 75 kDa, as validated in [41] and RANBP9 at ~ 115 kDa. Raw blots for each IP are provided in Supplementary Fig. 5 and western blots of input homogenate levels of total tau, pT217 tau, p62/SQSTM1, WDR26 and RANBP9 are provided in Supplementary Fig. 6

Co-IP of pT217 tau showed strong enrichment of SQSTM1 in all AD cases (Fig. 4b), with the strongest detections of SQSTM1 in the cases with the most tau (Supplementary Fig. 6). There was also evidence of a strong interaction between SQSTM1 in pT217 tau in PiD, and to a lesser extent in CBD and PSP. A weak interaction between pT217 tau and SQSTM1 was also observed in controls, suggesting SQSTM1 interacts with physiological tau in typical autophagolysosomal processes [7]. Validation co-IPs enriching for total tau and probed for WDR26 showed significant enrichment of WDR26 for all AD cases, indicating an interaction (Fig. 4b). There was no evidence of an interaction between tau and WDR26 in control cases, suggesting this interaction is specific to disease-associated tau (Fig. 4b). There were observable but faint evidence of an interaction between tau and WDR26 in PiD and PSP, but not CBD, suggesting WDR26 may interact with tau in select primary tauopathies in addition to AD. Validation co-IPs enriching for total tau and probed for RANBP9 also demonstrated enrichment for all three AD cases, indicating an interaction (Fig. 4c). Weak enrichment was also observed in one control case (CTRL2). No enrichment was observed in tauopathy cases.

**Fig. 5:**
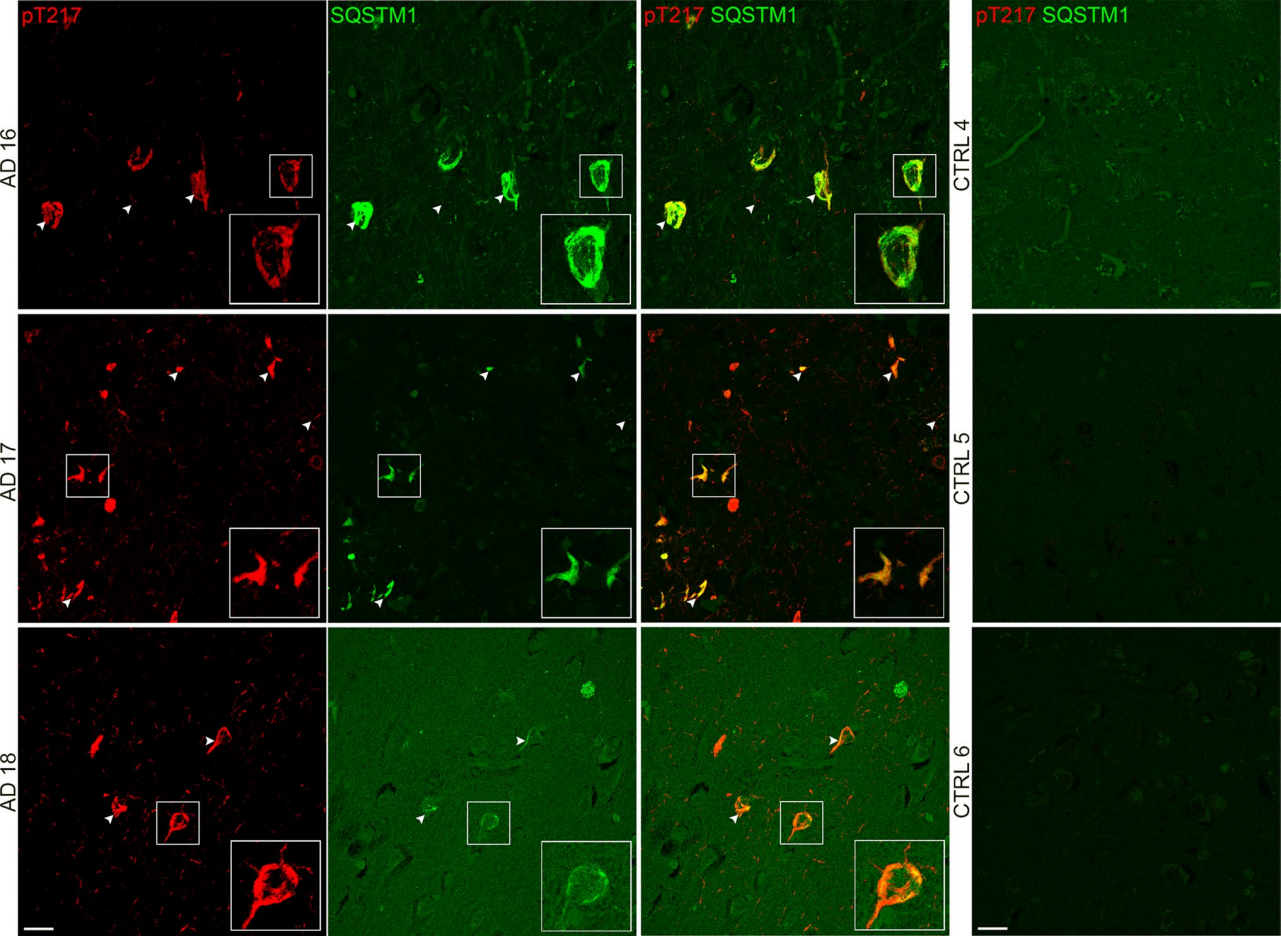
p62/SQSTM1 colocalises with pT217 tau in human post-mortem frontal cortex. Immunofluorescent micrographs of *n* = 3 AD and *n* = 3 control post-mortem brain sections co-stained for pT217 tau (red) and p62 (green). Boxes mark inset regions. Arrows mark instances of colocalisation. Scale bar = 20 µm. Negative controls (no primary antibody) are included in Supplementary Fig. 8

**Fig. 6:**
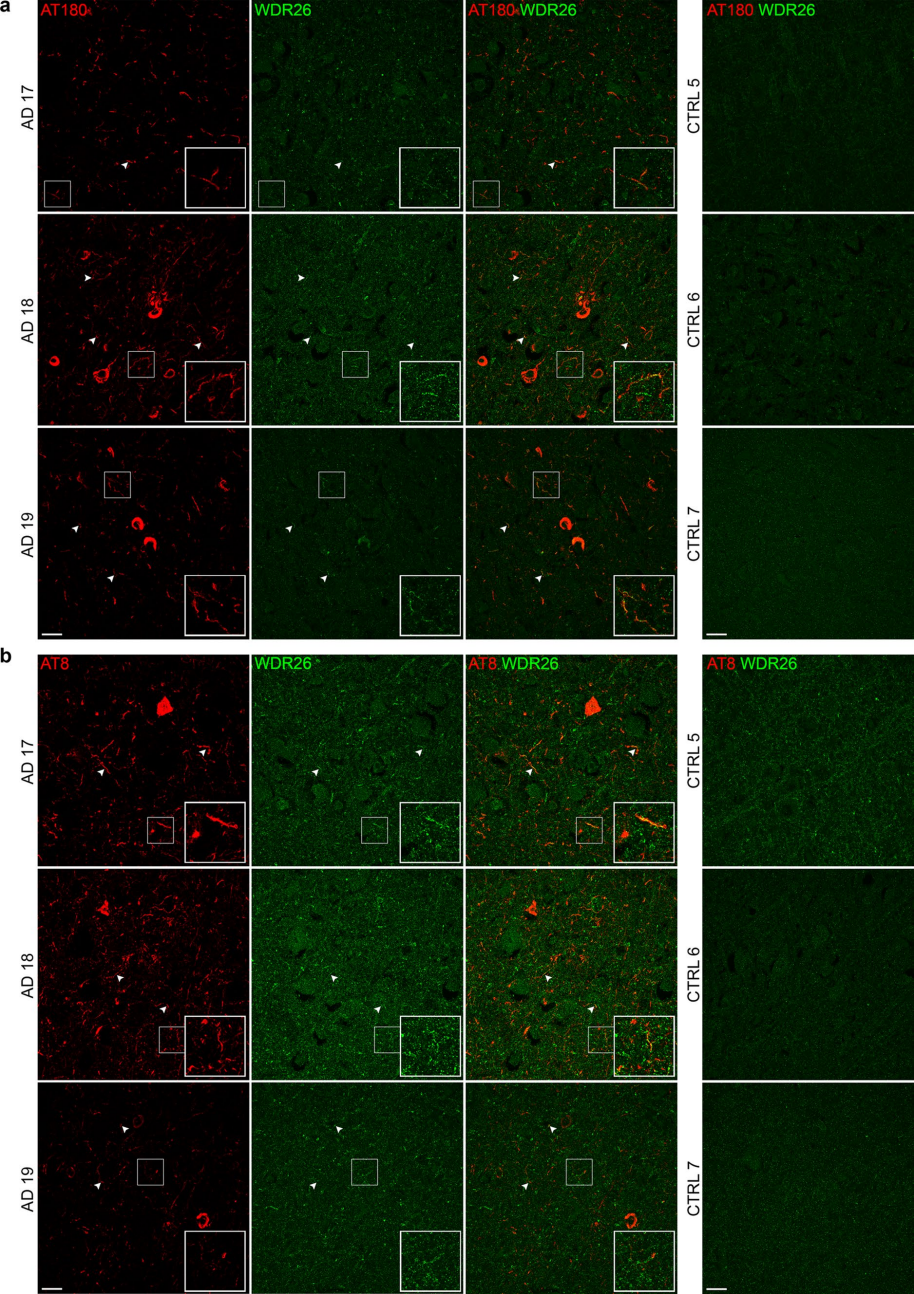
WDR26 colocalises with pTau in human frontal cortex. Immunofluorescent micrographs of *n* = 3 AD and *n* = 3 control post-mortem brain sections co-stained for **a** pT231 tau (red) and WDR26 (green) and **b** AT8 pTau (red) and WDR26 (green). Arrows mark instances of colocalization. Boxes mark inset regions. Scale bar = 20 µm. Negative controls (no primary antibody) are included in Supplementary Fig. 8

We next used immunofluorescence to determine if SQSTM1 and WDR26 colocalise with phosphorylated tau in human AD frontal cortex sections (*n* = 3 AD, *n* = 3 no-pathology controls). We observed strong colocalisation of pT217 tau and SQSTM1 (Fig. 5) in AD cases. Colocalisation was observed in neurofibrillary tangles, dystrophic neurites and neuropil threads. Interestingly, we observed several cases of dysregulated SQSTM1 that appeared to be encased in pT217 tau (Supplementary Fig. 7). Controls showed little to no SQSTM1 or pT217 tau staining. We next confirmed the interaction between WDR26 and phosphorylated tau by co-staining WDR26 with antibodies that detect pS202/pT205 tau (AT8) and pT231 tau (AT180). Species availability of WDR26 antibodies prevented us from using pT217 tau antibodies for this experiment. The distribution of WDR26 appeared different in AD and control cases: while WDR26 showed punctate staining in both AD and controls, the puncta in AD were typically larger and often clustered together in an organised manner within what appeared to be specific cells or processes (Fig. 6). In contrast, WDR26 staining in controls was diffuse, smaller puncta. WDR26 showed some positive colocalisation with both phosphorylated tau antibodies (Fig. 6a, b). Co-localisation between WDR26 and pTau occurred primarily in neuropil threads and dystrophic neurites throughout the frontal cortex with infrequent colocalisation of WDR26 in neurofibrillary tangles (Fig. 6a, b). As expected, control cases had minimal to no tau pathology.

**Fig. 7:**
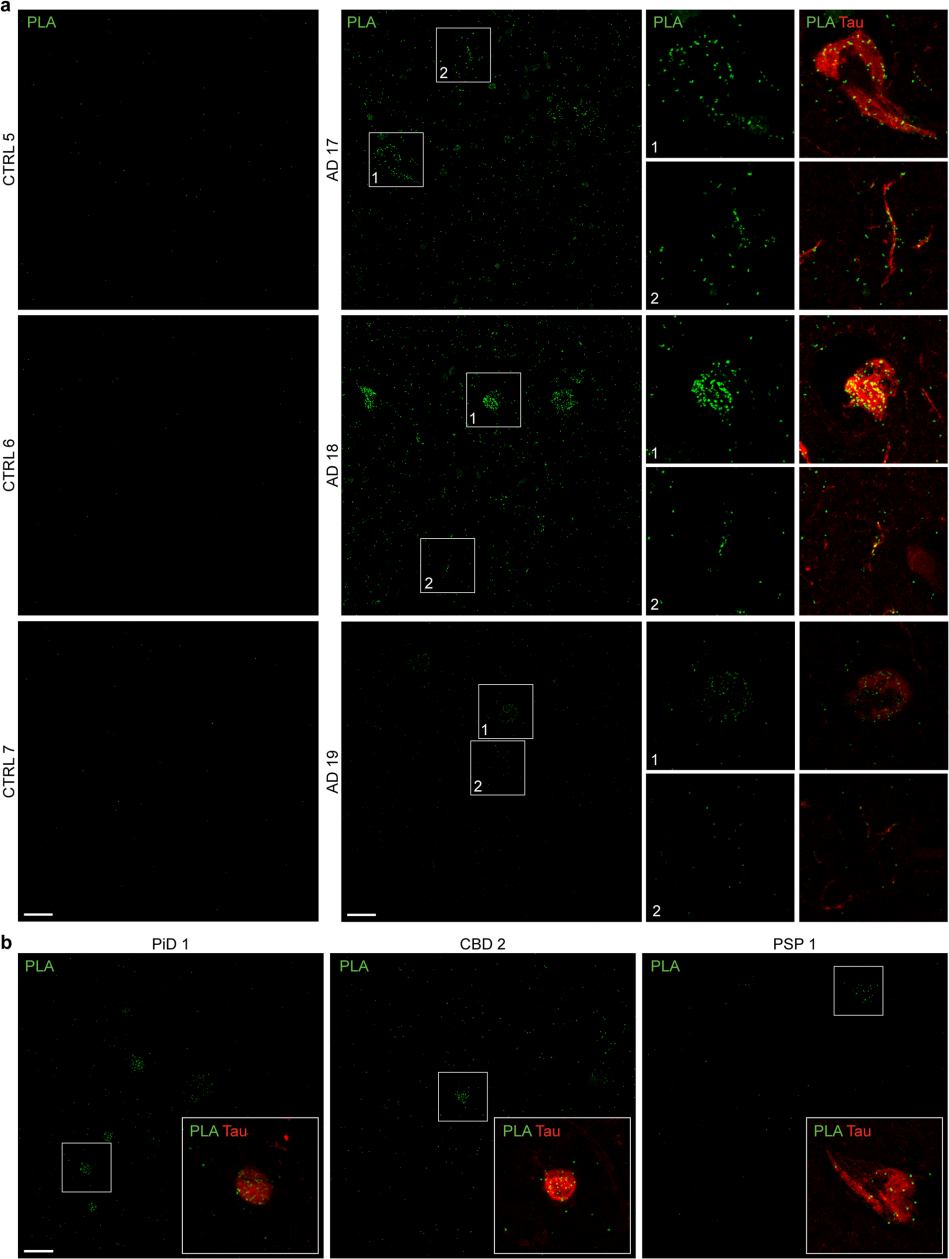
Proximity ligation assay detection of WDR26 and AT8 phosphorylated tau interactions. Immunofluorescent micrographs post-mortem brain sections stained for WDR26:AT8 tau PLA (green) and total Tau (red) in **a** *n* = 3 AD and control cases and **b** *n* = 1 CBD, PiD and PSP tauopathies. Scale bar = 20 µm. Boxes mark inset regions. Negative controls (no primary antibody) are included in Supplementary Fig. 8

Finally, we performed PLA of WDR26 and pS202/pT205 tau (AT8) to determine if WDR26 and pTau were located in close proximity (< 40 nm) in human brain tissue, potentially indicating an interaction. The control cases were largely absent of signal (Fig. 7a). In contrast, we observed frequent PLA-positive signal in AD cases often colocalising with neurofibrillary tangles, dystrophic neurites and neuropil threads (Fig. 7a). Additional diffuse PLA-positive signal was also frequently observed outside of tau aggregates, at a rate much higher than in controls. PLA-positive signal was also observed in Pick’s disease, CBD and PSP, often colocalising with neuronal phosphorylated tau aggregates in each disease (Fig. 7b). Notably, the PLA positive signal in tauopathies was less frequent than that observed in AD, supporting our co-IP results that suggested a stronger interaction between WDR26 and tau in AD than in tauopathies.

## Discussion

Here we report the first study of the pT217 tau interactome in human AD brain tissue, identifying 23 *bona fide* interactors of pT217 tau in AD. Additionally, we show that tau enriched using antibodies recognising individual phosphorylated epitopes have unique phosphorylation profiles, with pT217 tau phosphorylated at a lower number of residues in comparison to PHF1-immunoreactive tau. Based on these results, we hypothesise that pT217 and other residues we identified as consistently phosphorylated at high levels in all pools of enriched tau examined (e.g. T181, T231, S235) are early phosphorylation events in tau dysregulation. These findings concomitantly correlate with fluid biomarker results that identified the same phosphorylated tau species as early biomarkers of AD [3, 4, 38].

Of the 23 pT217 tau interactors identified, five proteins belong to the CTLH E3 ubiquitin ligase complex (ARMC8, GID8, MAEA, RANBP9 and WDR26). Interestingly, the four significant interactors identified for pT231 tau were also all CTLH E3 ligase subunits (ARMC8, GID8, RANBP9 and WDR26), despite the poor enrichment of tau. The interaction between CTLH E3 ligase subunits and tau phosphorylated at T217, T231 and S396/S404 was highly significant irrespective of *APOE* genotype. While the role of the CTLH E3 ligase complex has not been investigated in AD, previous proteomics-based interactome studies of AD brain tissue have consistently identified these same CTLH E3 ligase subunits as highly significant tau interactors [17, 22, 27, 52]. The role of one individual subunit, RANBP9, has been studied in the context of AD independent of its actions in the CTLH E3 ligase complex. RANBP9 enhances tau toxicity, promotes amyloid beta production and regulates synaptic protein levels in mouse and cellular models of AD [28, 29, 61]. As the effects of RANBP9 in these studies were assessed in isolation, it is not clear if the RANBP9 effects were via the CTLH complex or through CTLH complex-independent mechanisms. Interestingly, mutations of the CTLH complex component WDR26 (the most significant pT217 tau interactor) lead to intellectual disability, seizures and other neurological and developmental issues [19, 23, 49], suggesting that WDR26 has a fundamental role in brain development. Bulk tissue proteomic studies show that the protein subunits of the CTLH complex are inconsistently dysregulated in the brain in AD: two subunits are significantly downregulated (ARMC8 and MAEA) and two are upregulated (WDR26 and YPEL5) [5, 26]. This suggests that the complete CTLH E3 ligase complex is not broadly differentially expressed in disease, but there may be significant alterations of specific subunits. Together, these studies suggest RANBP9 and WDR26 likely have important functions in the brain and may play a role in disease toxicity through interactions with tau. Further exploration of WDR26 and the CTLH E3 ubiquitin ligase are important to determine if this complex is involved in tau clearance in AD, and if this role is limited to AD or also occurs in other tauopathies.

Notably, CTLH E3 ligase subunits only appear to interact with pathological forms of phosphorylated tau in human brain tissue. These same protein subunits do not significantly interact with tau in cell models, or animal models of disease, or in control human brain tissue [27]. This is particularly highlighted in a previous study that showed that WDR26 was one of the most significant tau interactors in AD compared to control cases [22]. Our validation studies support these results, showing that WDR26 appears to interact with tau only in disease but not in control brain tissue. The interaction between WDR26 and tau was much stronger in AD cases than in PSP, CBD or PiD, suggesting that this interaction could be more prevalent in AD. In contrast, the interaction between tau and SQSTM1 was also observed in control cases, albeit with less relative enrichment compared to AD or PiD. The interaction between RANBP9 and pTau showed a slightly different pattern to that observed for WDR26: while the strongest interaction between RANBP9 and pTau was still observed in AD cases, we also observed an interaction in one control case but no interaction in Pick’s disease, CBD or PSP. This suggests that the RANBP9-tau interaction may also be more prevalent in AD than in tauopathies. PLA results suggested that WDR26 likely interacted with pTau in distinct puncta in a subpopulation of neurofibrillary tangles and neuropil threads as well as in locations distinct from tau aggregates. This localization pattern, coupled with the lack of concentrated WDR26 in neurofibrillary tangles in our immunostaining studies, suggests that the WDR26-pTau interaction may preferentially occur with newly dysregulated tau prior to its recruitment into larger tau aggregates.

The CTLH E3 ubiquitin ligase is a large, modular and highly conserved complex with orthologues from yeast (GID complex) to humans, indicating an important biological role [33]. This complex is composed of a heterodimer of ring proteins (MAEA and RMND5) that form the base of the complex to which GID8, ARMC8 and RANBP9 bind. The isoform of ARMC8 appears to affect the binding of GID4 to the complex. The complex can exist either as a monomeric conformation or large hetero-oligomeric forms. This supramolecular formation appears to be controlled by the addition of WDR26 or MKLN1 which produce complexes with distinct substrate pools [15, 33, 47, 56]. The addition of each of these components appears to be dependent on RANBP9 [56]. The binding of WDR26 or GID4 appears to drive the substrate specificity of the CTLH complex [30, 39] and provide functional diversity to the complex. The human targets of the CTLH complex are not well documented but include its own component MKLN1 and the transcription factor HBP1 [30, 34] which suggest both autoregulation of its own assembly and possible broad activity by targeting transcription factors. While WDR26 is a consistent phosphorylated tau interactor, MKLN1 does not interact with any type of tau in our own data or previously published AD interactome studies [17, 22, 52]. This suggests that the specific CTLH complex that engages tau in AD is the subtype that contains WDR26, and not MKLN1. Further work is required to determine the functional consequences resulting from the interaction between the CTLH complex and phosphorylated tau, as while it could promote degradation of tau, it could potentially enhance tau aggregation, as ubiquitination of tau at specific residues can promote aggregation [43–45, 55, 60].

The protein interactors enriched in pT217 co-IP were largely consistent with those enriched by PHF1 co-IP. The seven unique interactors to pT217 tau were biased towards protein degradation pathways compared to PHF1 enriched tau interactors. However, the overall order of enriched interacting proteins for pT217 or PHF1 co-IPs were weakly correlated (Spearman’s rho = 0.303) suggesting that pT217 tau may in fact have biases in interactions that would likely lead to small deviations in interacting partners.

Some limitations to our study should be considered. For example, the number of tau interactors identified is highly dependent on antibody specificity and the efficiency of co-IP. The failure of pT231 (AT180) antibody to significantly enrich tau highlights this issue. The smaller number of tau interactors for pT217 tau in comparison to PHF1-immunoreactive tau was likely due to the antibody efficiency rather than evidence of pT217 tau interacting with fewer proteins in the brain. Similarly, the lack of *APOE* genotype effect on the pT217 interactome may be due to lower efficiency of pT217 antibody in enriching tau, given that this result contrasts with our previous study that reported significant differences in the PHF1-immunoreactive tau interactome in *APOE* ε3/ε3 versus *APOE* ε4/ε4 carriers [52]. Alternatively, *APOE* genotype may only impact tau interactions in later stages of dysregulation where it alters the subcellular localisation of PHF1 positive tau. As such, our interpretation is that we have identified only the most significant pT217 tau interactors. Additionally, it is unknown if the tau interactors identified here are restricted to only those present in the soluble fractions. Our data suggest at least some oligomeric and high-molecular weight aggregate species are enriched by Co-IP, as the western blots show a large smear extending beyond the typical molecular weight of tau. To overcome these issues future studies to fully characterise pT217 and pT231 interactions (and especially to tease apart *APOE* genotype effects) would require larger cohorts and perhaps alternative high-throughput approaches such as proximity ligation (BAR, APEX) coupled with mass-spectrometry. Future studies assessing the interactome of pT217 and other early phosphorylation events of tau in preclinical AD cases would be important for confirming the CTLH complex interaction is an early disease event and an early event in tau dysregulation. Furthermore, it is important for future studies to determine the physiological outcomes of the tau-CTLH complex interaction. This could be assessed by altering levels of CTLH complex proteins in cell culture models that express tau aggregates. This would help to decipher if tau is being turned over by the CTLH complex.

In conclusion, we have reported the phosphorylation profile and interactome of pT217 tau in human brain tissue. We hypothesise that pT217 tau may be a less mature pathological form of tau in comparison to PHF1-immunoreactive tau based on their differing phosphorylation profiles. We have identified a significant interaction between multiple types of phosphorylated tau and the CTLH E3 ubiquitin ligase in AD, warranting further study examining the role of this new complex in AD.

## Supplementary Information

Below is the link to the electronic supplementary material.

Supplementary file1 Supplementary Fig. 1 Phosphorylation profiles of tau enriched by pT217, pT231 and PHF1 co-IP. This shows figure 1 additionally including phosphorylated residues detected in co-IPs of pT231 as well as pT217, and PHF1 tau (S396, S404) mapped against full length 2N4R tau. Green flags represent serine phosphorylation and orange represent threonine phosphorylation. Colour shade of nodes represents the binned average intensity (i.e. 21 = 21-21.99, 30 = 29-30) for each residue. Node size correlated to the number of samples positive for phosphorylation. Statistics shown are group-wide results. ‘ns’ = not significant. (EPS 1006 KB)

Supplementary file2 Supplementary Fig. 2 pT231 co-IP results. a Western blot of pT231 co-IP demonstrated moderate enrichment for phosphorylated tau at T231 in comparison to control IgG co-IP (n = 10 AD cases: n = 5 APOE ε3/ε3 and n = 5 APOE ε4/ε4). b pT231 tau interactors identified by affinity purification mass-spectrometry (SAINT score ≥ 0.65). c Venn diagram showing the intersections between pT217 and pT231 interacting proteins. d Over-represented gene ontology biological process terms for the four significant pT231 interacting proteins. (EPS 4608 KB)

Supplementary file3. Supplementary Fig. 3 AP-MS total brain homogenate characterization. Unedited western blot results of a enrichment of phosphorylated tau following pT217 tau co-immunoprecipitation of all samples used in AP-MS. Probed with rabbit pTau S199/202 (Thermo, #44-768G, 1:500) b pT217 abundance in input total brain homogenates used for AP-MS c enrichment of phosphorylated tau following pT231 tau co-immunoprecipitation of all samples used in AP-MS. Probed with PHF1 tau (provided by Peter Davies) d pT231 abundance in input total brain homogenates used for AP-MS. (EPS 11070 KB)

Supplementary file4 Supplementary Fig. 4 pT231 co-IP APOE genotype sub-analysis results. a pT231 tau interactors identified in ε3/ε3 patients by mass-spectrometry (SAINT score ≥ 0.65) of pT231 enriched fractions. b pT231 tau interactors identified in ε4/ε4 patients by mass-spectrometry (SAINT score ≥ 0.65) of pT231 enriched fractions. c Overlap of pT231 interactors enriched in APOE ε3/ε3 and ε4/ε4 patients, Jaccard Index = 0.15, Fisher’s exact test, p = 1.41x10-9. d Enrichment map of gene ontology cellular compartments over-represented from pT231 interactors in APOE ε3/ε3 or ε4/ε4 sub-groups. e Venn diagram showing the overlap between pT217 and pT231 interactors in patients with APOE ε3/ε3 genotype. f Venn diagram showing the overlap between pT217 and pT231 interactors in patients with APOE ε4/ε4 genotype. (EPS 2038 KB)

Supplementary file5 Supplementary Fig. 5 Validation co-IP western blot raw images. Raw western blot images of a pT217 validation co-IP probing for p62/SQSTM1 (Abcam, ab56416 1:000) and b total tau co-IP (Tau-5, Abcam ab76128) probed for WDR26 (Abcam, ab85962, 1:500). c total tau co-IP (Tau-5, Thermo MA512808) probed for RANBP9 (Abcam ab52605, 1:1000) The dark bands in b and c at ~50 kDa and ~25 kDa are secondary cross-reactions with the heavy and light chains of the antibodies used for co-immunoprecipitation. (EPS 28289 KB)

Supplementary file6 Supplementary Fig. 6 Validation co-IP total homogenate input characterization. Raw western blot images of total homogenate protein used in validation co-IPs probed for a pT217 (Thermo #44-744, 1:1000), b p62/SQSTM1 (Abcam, ab56416 1:000), c Total tau (Thermo MA5-12808, 1:1000), d WDR26 (abcam ab85962, 1:500) and e RANBP9 (Abcam ab52605, 1:1000). (EPS 22770 KB)

Supplementary file7 Supplementary Fig. 7 Examples of SQSTM1 structures surrounded by pT217 tau. High zoom micrograph of human frontal cortex sample AD 15 stained for pT217 (red) and SQSTM1 (green). Main image is a max projection. Examples of SQSTM1 structures are inset a and b with orthogonal views of 48 stacks. Scale bar = 20 µm, z-steps were set to 0.125 µm. (TIF 11014 KB)

Supplementary file8 Supplementary Fig. 8 IHC and PLA negative controls. Regions of interest taken from slide scans of negative controls for a p62/SQSTM1 (green) and pT217 (red) co-stain, b WDR26 (green) and AT8 (red) co-stain, c WDR26 (green) and AT180 (red) co-stain and d WDR26:AT8 PLA (green) and total tau (red) co-stain. Autofluorescence (AF) detected in the empty λ561 channel is shown in greyscale. Scale bar is 200 µm. (TIF 32740 KB)

Supplementary file9 Supplementary tables 1-14 (XLSX 6370 KB)

## Data Availability

The mass spectrometric raw files are accessible at https://massive.ucsd.edu under accession MassIVE MSV000096377 and at www.proteomexchange.org under accession PXD057755.
