# Supplementary figures and images for: The interactome of tau phosphorylated at T217 in Alzheimer’s disease human brain tissue
Source: Acta Neuropathol. 2025 May 3;149(1):44. doi: 10.1007/s00401-025-02881-8 (PMC12049313; doi:10.1007/s00401-025-02881-8)

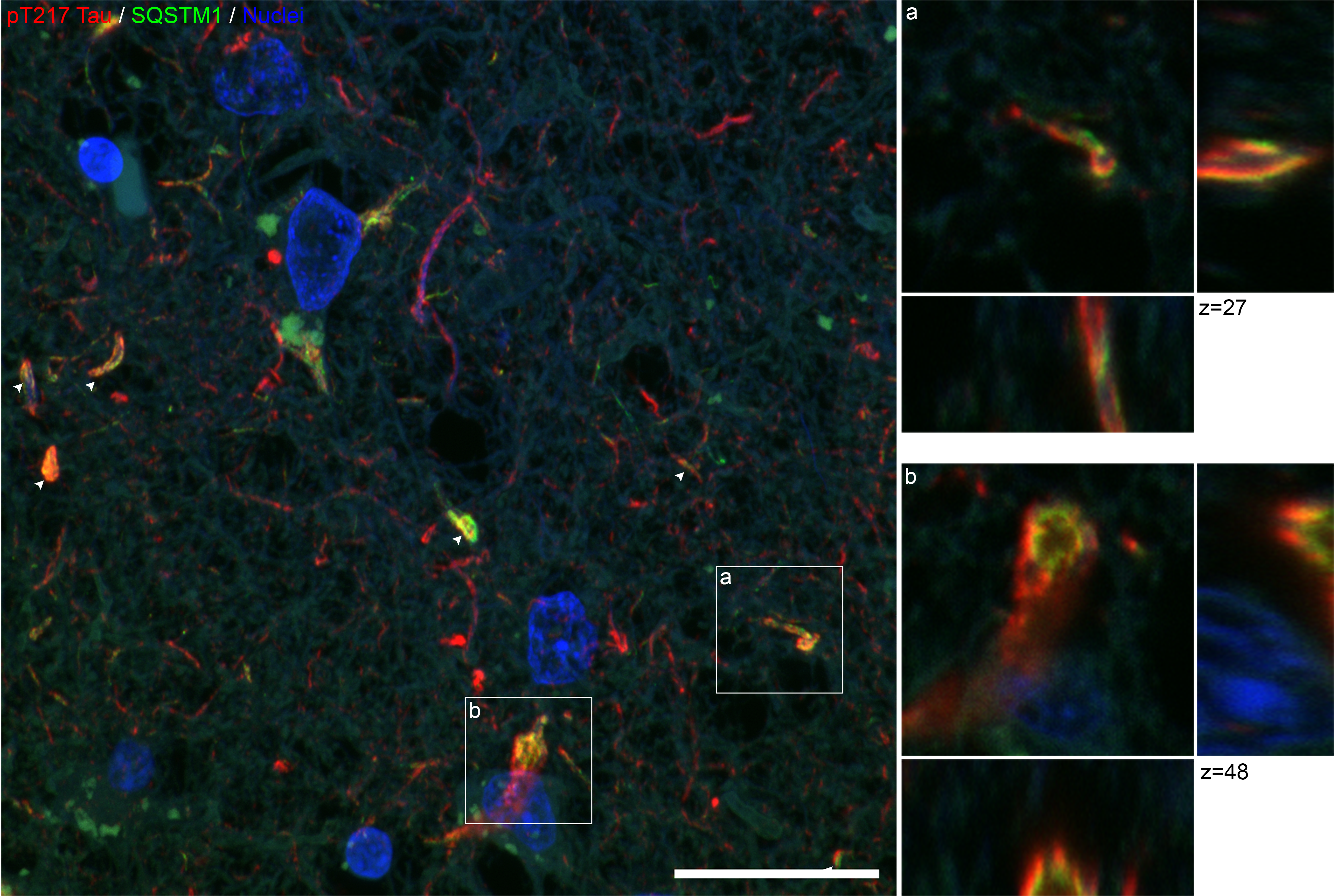

Supplement: Supplementary file 7 — Supplementary file7 Supplementary Fig. 7 Examples of SQSTM1 structures surrounded by pT217 tau. High zoom micrograph of human frontal cortex sample AD 15 stained for pT217 (red) and SQSTM1 (green). Main image is a max projection. Examples of SQSTM1 structures are inset a and b with orthogonal views of 48 stacks. Scale bar = 20 µm, z-steps were set to 0.125 µm. (TIF 11014 KB) [file 401_2025_2881_MOESM7_ESM.tif]

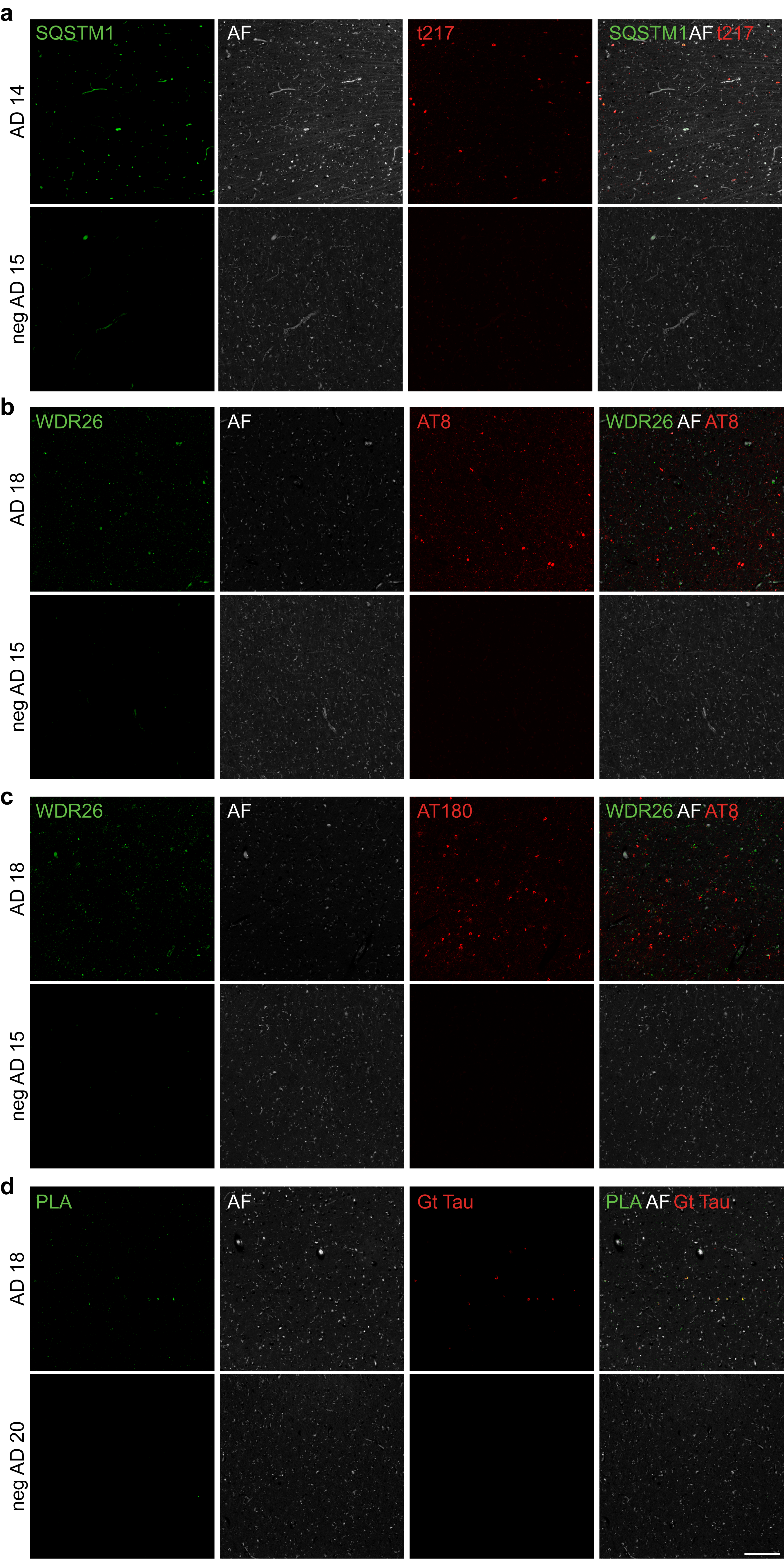

Supplement: Supplementary file 8 — Supplementary file8 Supplementary Fig. 8 IHC and PLA negative controls. Regions of interest taken from slide scans of negative controls for a p62/SQSTM1 (green) and pT217 (red) co-stain, b WDR26 (green) and AT8 (red) co-stain, c WDR26 (green) and AT180 (red) co-stain and d WDR26:AT8 PLA (green) and total tau (red) co-stain. Autofluorescence (AF) detected in the empty λ561 channel is shown in greyscale. Scale bar is 200 µm. (TIF 32740 KB) [file 401_2025_2881_MOESM8_ESM.tif]
